# Targeting ocular malignancies using a novel light-activated virus-like drug conjugate

**DOI:** 10.1016/j.aopr.2024.12.001

**Published:** 2024-12-03

**Authors:** Sen Ma, Ruben V. Huis In't Veld, Elisabet de los Pinos, Ferry A. Ossendorp, Martine J. Jager

**Affiliations:** a Department of Ophthalmology, Leiden University Medical Center (LUMC), Leiden, the Netherlands; b Department of Radiology, Leiden University Medical Center (LUMC), Leiden, the Netherlands; c Department of Immunology, Leiden University Medical Center (LUMC), the Netherlands; d Aura Biosciences, Inc., Cambridge, MA, USA

**Keywords:** Eye, Oncology, Melanoma, Virus-like particles, Targeted therapy, Photodynamic therapy

## Abstract

**Background:**

Targeted therapy is a promising approach to improve the treatment of tumors, including ocular malignancies. Current therapies, such as radiotherapy and surgery, often lead to serious damage to vision or to loss of the eye. New approaches have examined nanoparticles for use as targeted delivery vehicles for drugs. A newly-developed virus-like drug conjugate is a promising nanoparticle with a defined target: the novel virus-like particle-photosensitizer conjugate Belzupacap sarotalocan (Bel-sar, previous name AU-011).

**Main text:**

In this review, we summarize the application of this novel light-activated virus-like particle conjugate in pre-clinical and clinical studies and discuss its potential to treat ocular malignancies, such as uveal melanoma and conjunctival melanoma. We furthermore discuss the combination with immunotherapy and its application on pigmented and non-pigmented tumors as well as its effect on macrophage polarization, which is important to achieve effective results in immunotherapy.

**Conclusions:**

Belzupacap sarotalocan (Bel-sar) is a promising targeted drug carrier that enhances tumor-specific delivery and minimizes off-target effects. Its photodynamic therapy effectively treats pigmented and non-pigmented tumors while inducing immunogenic cell death through DAMP exposure, triggering local and systemic immune responses. Combining Bel-sar PDT with immunotherapy improves efficacy in preclinical models, warranting further clinical investigation.

## 1 Introduction

Targeted delivery has long been a challenging goal for improving the treatment of malignancies. Especially for ocular tumors, traditional treatment including radiotherapy, enucleation and excision, may lead to serious visual damage or even loss of the eye. Ocular malignancies constitute rare diseases but are often sight-threatening and lethal. Here, we review a novel targeted approach for ocular cancers using a light-activatable virus-like drug conjugate known as Bel-sar (belzupacap sarotalocan) for which recent studies indicate a promising clinical potency.

Uveal melanoma (UM) is the most common primary ocular malignancy in adults while conjunctival melanoma (CJM) is a rare ocular surface tumor.^1^, ^2^, ^3^ Uveal melanoma can occur in the iris, the ciliary body and the choroid. Improvement in local treatments has helped to spare the eye, but surgery and irradiation still often lead to structural damage and do not prevent the formation of metastasis; even after successful treatment, 50% of UM and 30% of CJM patients still may develop metastasis in distant organs like liver, lungs or lymph nodes.^4^, ^5^, ^6^ When metastases are clinically evident, prognosis for patients with UM and CJM drastically decreases to a median survival of approximately 6.3 months, and 24 months, respectively.^7^, ^8^, ^9^

Immunotherapy such as with immune checkpoint inhibitors has improved the survival of patients with some types of cancer, such as colon cancer, lung cancer and cutaneous melanoma.^10^, ^11^, ^12^ Interestingly, CJM often has a similar genetic background as cutaneous melanoma, and case reports indicate that CJM patients can benefit from immune checkpoint inhibitor treatment.^3^ Unfortunately, immune checkpoint inhibitors have not improved the survival of patients with UM metastases.^13^^,^^14^ The main reasons for the lack of efficacy may be related to the low mutational burden of UM and the immune privilege of the eye, which may not only inhibit local immune responses but also confer immune resistance on metastases.^15^^,^^16^ This may be overcome by a stronger induction of local immune responses, for instance, by using multiple immune checkpoint inhibitors or by using additional treatments such as Photodynamic therapy.

Photodynamic therapy is a clinically-applied, minimally-invasive tumor ablation method that involves the administration of a photosensitizer and irradiation with light which is often in the red-light spectrum. In the presence of oxygen, the energy of the light is transferred to a photosensitizer inducing the production of reactive oxygen species (ROS); ROS can directly kill tumor cells or indirectly, by affecting the tumor vasculature.^17^^,^^18^ In addition, cell death after photodynamic therapy can initiate immunogenic cell death, a mode of cell death that is often accompanied by the exposure and release of damage-associated molecular patterns (DAMPs).^19^^,^^20^ This then initiates a local inflammatory response at the treated site, which can develop into systemic anti-tumor immunity, providing long-term tumor growth control.^21^ Although PDT at low settings may not induce immunogenic cell death, the current results that display a DAMP response and subsequent acture inflammation in the tumor are based on PDT settings that were optimized to indice ICD.^22^ Photosensitizers, such as verteporfin, have been used as an alternative treatment to radiotherapy for primary UM to preserve vision.^23^^,^^24^ Unfortunately, there has been limited clinical benefit, with incomplete destruction of the tumors that then resulted in survival of tumor cells, followed by rapid regrowth and tumor progression.^24^ This may be due to tumor pigmentation, poor biodistribution of the photosensitizer or a lack of tumor-specific targeting. The treatment may be improved by using a novel carrier system with the ability to specifically target malignant cells that can be loaded with a large number of photosensitizer molecules or combined with other cytotoxic modalities.

**Fig. 1:**
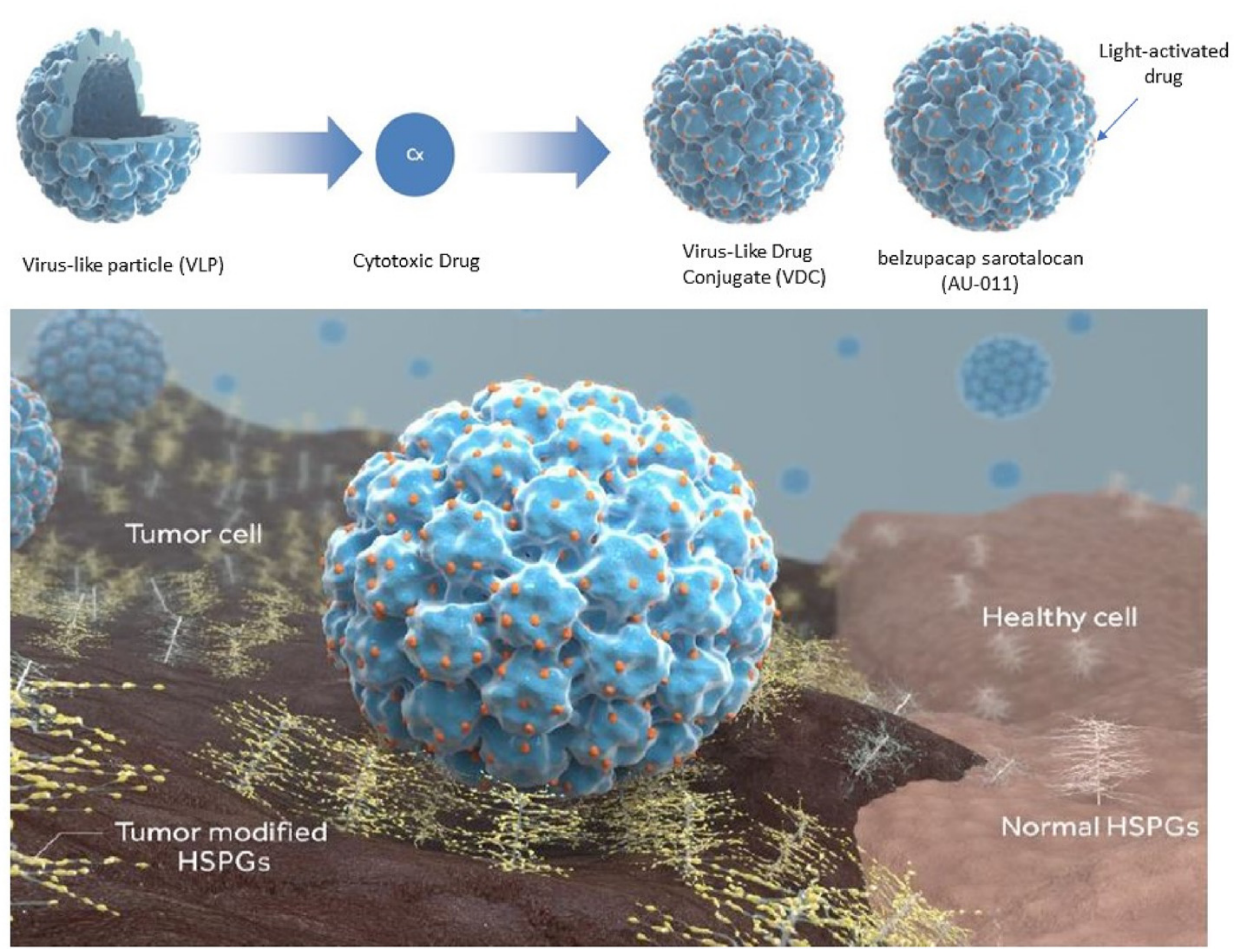
The structure of the virus like particle (VLP), the virus-like drug conjugate (VDC) and Belzupacap sarotalocan (Bel-sar, AU-011), and its targeting tumor mechanism. The VLPs are derived from the capsid of Human Papillomavirus 16 (HPV16), without its genome. The VLPs can serve as a delivery vehicle to load drugs, such as the phthalocyanine photosensitizer, creating a virus-like drug conjugate. Bel-sar consists of an HPV16-derived VLP conjugated to around 200 molecules of phthalocyanine. VDCs selectively bind to tumor-associated heparin sulfate proteoglycans (HSPGs).

New carrier systems for photosensitizers include antibodies, liposomes and nanoparticles, that may facilitate a more favorable biodistribution. Most of the work has focused on liposomal and polymer-based nanoparticles, while new research analyses the use of pseudovirions and virus-like particles (VLPs). Recently, Human papillomavirus (HPV) VLPs have been used as carriers for targeted therapy, as VLPs preferentially bind and infect tumor cells by binding to heparin sulfate proteoglycan (HSPGs) molecules on the cell surface.^25^^,^^26^ These VLPs are being used as delivery vehicles, loaded with a large number of phthalocyanine photosensitizer molecules and are known as virus-like drug conjugates (VDC), named Bel-sar (previously known as AU-011) (Fig. 1). Treatment with light-activated Bel-sar is currently being used as the first-line treatment of small choroidal melanoma or indeterminate choroidal pigmented lesions in order to inhibit tumor growth early on, while preserving vison and the eye of the patient (Phase 3 trial, NCT06007690). As Bel-sar specifically binds to malignant cells by multivalent binding of HSPGs, this results in a high deposition of photosensitizer in the tumor cells. There is minimal toxicity of Bel-sar itself without irradiation. Initial clinical investigations evaluated Bel-sar with either suprachoroidal or intravitreal administration in patients with small choroidal melanomas. Pre-clinical data demonstrated that suprachoroidal administration of Bel-sar in leads to optimal biodistribution of Bel-sar in the choroidal tumors. These characteristics make it possible to induce in situ tumor ablation with minimal side effects, accompanied with immunogenic cell death which could trigger a systemic immune response to potentially prevent metastases. In a preclinical study, Bel-sar and light were applied on tumors derived from UM cell line 92.1 in an immunosuppressed intra-ocular UM model in rabbits and it was shown that the combination of AU-011 and light proved effective in killing the tumor, while sparing the retina and adjacent ocular structures.^27^ We postulated that one should investigate multiple cell lines, as differences in sensitivity might occur, based on genetic or pigmentation differences occurring in individual patient's tumor types. Furthermore, one may consider using Bel-sar alone or in combination with immune checkpoint inhibitors. We report on our findings and summarize our work on the application of Bel-sar in ocular malignancies.

## 2 Virus-like particle conjugated to photosensitizer for cancer therapy

Virus-like particles (VLPs) are self-assembling nanoparticles (NPs) derived from the capsid proteins of viruses, without containing their natural genome. The definite surface chemistry, uniform size, and stability make the VLPs an efficient delivery vehicle to deliver payloads such as chemotherapeutic drugs, siRNA, DNA, proteins, peptides and photosensitizers to cells.^28^, ^29^, ^30^, ^31^, ^32^ Most nanoparticles deliver drugs through encapsulation, which protects the drug from degradation and ensures targeted delivery to specific tissues or cells. In contrast, VLPs used as delivery systems are conjugated with molecules such as antibodies, ligands, or drugs to enhance their targeting capabilities. The advantages of VLPs are that they can be conjugated with small molecules; they deliver high numbers of payloads in a single VLP; they selectively recognize targeted cells and have minimal off-target effects; they release or activate their cargo in response to certain stimuli.^33^ Virus-like particle-mediated photodynamic therapy has been used to treat malignancies, such as prostate cancer, breast cancer and melanoma.^34^, ^35^, ^36^ Hemagglutinating virus of Japan envelope (HVJ-E) was loaded with Protoporphyrin IX photosensitizer to enhance the uptake of PpIX and cytotoxicity of photodynamic therapy in vitro by specific binding of the sialic acid receptor on prostate cancer cells.^34^ A phage particle modified with pyropheophorbid-a photosensitizer was used to selectively target breast cancer cells in vitro by binding peptides.^35^ Wen and co-workers^36^ have utilized Nanoparticle cowpea mosaic virus (CPMTV), which interestingly has dual specificity for the immunosuppressive subpopulation of macrophages and simultaneously targets cancer cells. CPMTV served as delivery vehicle for zinc ethynylphenyl porphyrin photosensitizer to enhance photosensitizer accumulation in M2 macrophages and cancer cells in vitro. These VLP vehicles have been shown to enhance the accumulation of PS in vitro in one type of cancer, but still lack truly specific tumor targeting in vitro and in vivo.

Bel-sar is a virus-like drug conjugate (VDC) composed of VLPs from modified Human papillomavirus (HPV), which have been conjugated to the photosensitizer phthalocyanine (Fig. 1).^26^^,^^27^ The HPV-derived VLPs can specifically target malignant cells by binding to tumor modified glycosaminoglycans on heparan sulfate proteoglycans (HSPGs). These HSPGs are preferentially expressed and modified on tumor cells and not on the cell surface of normal cells.^37^ The conjugated phthalocyanine photosensitizer can be activated with lasers with a wavelength of 690 ​nm which can penetrate relatively deep into tissues, and in the presence of oxygen, leads to the generation of reactive oxygen species (ROS), inducing tumor cell destruction.

## 3 Targeting ability of HPV VLPs

The virus-like particles derived from HPV specifically target tumor cells in a similar manner to HPV during the infection process.^25^ HPV cannot bind directly to epithelial cells of intact tissues, and HPVs primary binding occurs through heparan sulfate proteoglycans (HSPGs).^26^ Only when there is exposure of HSPGs on e.g. the basement membrane or extracellular matrix can they serve as a binding site for HPV. This then induces various conformational changes of the HPV, leading to epithelial cell surface receptor binding. Following this binding, HPVs are engulfed into the cytoplasm. Kines et al. showed that HPV capsids (VLPs) have the same restricted tropism to epithelial cells as the virus itself, and preferentially bind and infect tumor cells in vitro and in vivo, which process can be blocked by heparin or Iota-carrageenan.^25^ The HVP capsid cannot bind HSPG-deficient tumor cells. These findings indicate that tumor cell binding of HPV-VLPs is HSPG-dependent.

HSPGs are glycoproteins with a common characteristic that they contain one or more covalently-attached heparan sulfate chain, a type of glycosaminoglycan.^38^ HSPGs are ubiquitously present in tissues, mainly associated with the cell surface and the extracellular matrix. The role of HSPGs in cancer is not fully understood; yet, they are involved in a number of processes related to malignancy, such as tumorigenesis, angiogenesis and metastasis.^39^ Overexpression and modification in HSPG sulfation patterns on cancers are quite common, potentially making them an attractive target for HPV binding and infection.^40^, ^41^, ^42^ Both quantitative and qualitative changes in HSPGs are seen in parallel to progression of nevi to radial growth phase to vertical growth phase of cutaneous melanoma.^43^ Tumor cells display HSPG patterns that mimic the patterns normally found on the basement membrane or extracellular matrix but not the apical surface of normal epithelial or mesothelial cells. VLPs have been shown to directly bind and infect most tumor-derived cell lines in vitro and to have analogous tumor-specific properties in vivo.^25^ HPV VLPs therefore seem to be useful reagents to detect and potentially treat a remarkably broad spectrum of cancers.

## 4 Light activated bel-sar in ocular malignancies

### 4.1 Preclinical and clinical study

In the pre-clinical study in an intraocular orthotopic tumor model (92.1 ​cell line in a rabbit eye), an intravenous injection of Bel-sar and light treatment induced tumor necrosis with minimal damage to optical structures.^27^ It indicated that Bel-sar was mainly distributed to the eye tumor instead of other tissues of the eye. In another pre-clinical study using an orthotopic tumor model, a suprachoroidal injection induced a higher Bel-sar accumulation in the tumor compared with an intravitreal injection.^26^^,^^44^ In ongoing Phase II and III clinical trials, suprachoroidal administration of Bel-sar is being used to increase its distribution in the intraocular tumor and reduce any off-target effects. Recently, Aura presented the final result of a Phase 2 study (NCT04417530), which showed that Bel-sar treatment led to an 80% tumor control rate, 90% visual acuity preservation, and a highly favorable safety profile.^45^ More importantly, 80% of the patients had been at high risk for vision loss with tumors close to the fovea or optic disc if treated with radiotherapy, highlighting the potential for vision preservation with this novel class of drugs. The safety profile of Bel-sar was highly favorable in all participants regardless of the dose. These pre-clinical and clinical trials show the effectiveness and safety of Bel-sar as first-line treatment of small choroidal melanoma and indeterminate pigmented lesions (Table 1, Table 2).

**Table 1:** Cytotoxicity and DAMPs induced by Bel-sar treatment in cell lines from different origins (uveal melanoma, conjunctival melanoma, mouse).

Cell lines	Types	Main mutations	IC50	Increased fold of DAMPs		Reference
				CRT	HSP90	
OMM2.3	M-UM	GNAQ	15	9	1.4	^55^^,^^68^^,^^69^
Mel270	P-UM	GNAQ	17	11.5	1.9	
Mel285	P-UM	WT	18	8.6	1.4	
92.1	P-UM	GNAQ, EIF1AX	19	5.9	1.1	
OMM1	M-UM	GNA11	24	11.0	1.3	
MM66	M-UM	GNA11	25	9.0	1.4	
OMM2.5	M-UM	GNAQ	29	7.6	1.2	
MP38	P-UM	GNAQ, BAP1	33	4.0	2.1	
MP46	P-UM	GNAQ, BAP1	36	3.5	1.3	
MM28	M-UM	GNA11, BAP1	40	4.1	1.5	
CRMM1	P-CJM	BRAF	40	1.5	6.0	^3^^,^^67^
CRMM2	P-CJM	NRAS	31	1.6	5.5	
CM2005.1	M-CJM	BRAF	60	1.6	6.6	
B16F10 ​wt	Pigmented	WT	7	5.5	2.6	^66^
B16F10 ko	non-Pigmented	Tyrosinase	9	7.9	5.6	

P: primary; M: metastasis; UM: uveal melanoma; CJM: conjunctival melanoma; IC50 in pM, at fluence 25J/cm2, 600mW/cm2 fluence rate.

**Table 2:** Animal model and clinical trial of Bel-sar.

Cell lines	Tumor model	Treatment strategy	Immunostimulatory or outcome	Reference
92.1	Introaocular in rabbit	AU-011 PDT by intravenous injection	effective against the tumor and spared the retina or adjacent ocular structure	^27^
TC-1	subcutaneous in mice/rechallenge tumor	AU-011 combined with ICIs	DAMPs,Tumor-specific CD8+T cells	^73^
MC38	subcutaneous in mice/dual tumor	AU-011 combined with multiple ICIs	DAMPs, Dendritic cell maturation	^72^
B16F10 ​wt and ko	Subcutaneous in mice/paired tumor	AU-011 PDT in paired pigmented and non-pigmented tumor	DAMPs, Dendritic cell maturation, macrophage polarization	^66^
stage I	small UM (largest diameter <16 ​mm)	intravitreally administered, multiple ascending doses	Tolerated with maintenance of vision and tumor control	^97^
Stage II	Small UM in large group	Suprachoroidal administaiton with dose escalation study	High tumor control rate and visual acuity preservation	^45^
Stage III	multiple center, small to medal size UM	Suprachoroidal administaiton with high does	on going	^98^

The anti-tumor efficiency induced by photodynamic therapy is mainly dependent on the photochemical properties and biological effects of Bel-sar, its accumulation and biodistribution, the total energy for irradiation and more importantly, on the genetic, epigenetic, phenotypic, immunogenic cell death tumor profiles and tumor microenvironment.^46^ Defined tumor types, even with the same origin and sub-classification, still may have high heterogeneity, and this could contribute to a large discrepancy in photosensitizer uptake, cytotoxicity and immunostimulatory signals triggered by photodynamic therapy. The tumor-targeting ability and thereby efficient uptake of large numbers of photosensitizer-loaded HPV-VLPs contribute to adequate accumulation of Bel-sar and only small discrepancies between tumors in response to photodynamic therapy. In Table 1, we summarized the amount of cytotoxicity and expression of DAMPs induced by light activated Bel-sar in UM, CJM and B16F10 ​cell lines. Table 2 references the animal model and clinical trials of Bel-sar.

#### 4.1.1 Uveal melanoma

Uveal melanoma is the most common primary ocular malignancy in adults and is often lethal. UM could be a target of Bel-sar as the tumor has been shown to express HSPGs and has an increased sulfate expression in high grade disease.^47^^,^^48^ Furthermore, UM's that occur in the choroid are often directly visible in the back of the eye and therefore easy to treat with light. *In vitro* testing by Kines et al. showed that the cell line 92.1 is susceptible to killing by Bel-sar. When 92.1 ​cells were placed in the choroidal space of immuno-suppressed rabbits, they grew into treatable tumors that highly resemble the presentation of the human disease. Treatment with Bel-sar and light was able to kill large parts of the tumors, with minimal damage to the retina and adjacent ocular structures.^27^ However, not all UM cells are the same. It is known that loss of chromosome 3 and/or the presence of a BAP1 (BRCA1-associated protein 1) mutation in UM is associated with a high risk of metastasis formation, as well as with an increased density of tumor infiltrating lymphocytes and tumor associated-macrophages.^49^, ^50^, ^51^ The presence of tumor-infiltrating lymphocytes and macrophages may be involved in immune suppression. BAP1 is a ubiquitin carboxyterminal hydrolase that has been shown to be a tumor suppressor gene, is involved in cellular metabolism and DNA repair.^52^^,^^53^ Mutations are known to affect the efficiency of photodynamic therapy. In one study, a p53 mutation in a cancer clone affected the anti-tumor efficiency of photodynamic therapy in vitro and *in vivo*^54^. This may be due to the altered expression of tumor survival genes or an increase in DNA-repair processes. We hypothesized that the genetic background, such as the presence of a BAP1 mutation, might affect Bel-sar treatment in UM.

We therefore tested the binding and uptake ability of Bel-sar by a series of UM cell lines carrying different mutations.^55^ Bel-sar showed high binding levels in all ten UM cell lines tested, but preferentially bound to the BAP1-positive UM cell lines rather than the BAP1-negative ones, resulting in a higher IC50 of the BAP1-negative cell lines. Regardless of BAP1 mutation or other mutations in UM cell lines, the observation that the EC50 of Bel-sar treatment is in the picomolar range (15pM–40pM) indicates that UM cells are still sensitive to Bel-sar treatment regardless of their genetic profile (Table 1). When considering the immuno-suppressive microenvironment in BAP1-negative UM, it may be necessary to use a high dose of Bel-sar or multiple treatments to induce total tumor ablation. Treatment modes may need to be adjusted to the needs of each patient according to the individual characteristics of the UM in the clinic.

#### 4.1.2 Pigmentation

The major difference between melanoma and non-melanoma cancer cells is a unique subcellular organelle termed the melanosome, a lysosome-related organelle which is involved in melanin synthesis. In cutaneous melanoma, hyper-pigmentation decreases the outcome of radiotherapy and chemotherapy.^56^ In both CM and UM, a high pigmentation level is associated with bad prognosis.^57^^,^^58^ Melanogenesis may be involved in drug resistance in melanoma.^59^ Chen et al. explain how the different stages of the melanosome affect drug sensitivity. Stage I and II melanosomes, termed "pre-melanosomes", can trap and export cytotoxic drugs such as cisplatin. However, cytotoxic endogenous melanin in stage III melanosomes could enhance drug susceptibility.^60^ Interestingly, new evidence show that melanin can enhance the photochemical reaction by absorbing photons and transferring energy to the photosensitizer in a murine conjunctival melanoma model.^61^ Melanosomes are therefore a double-edged sword in the treatment of melanoma.

Pigmentation in UM and CM is known as a barrier for photodynamic therapy, as the pigment absorbs light across broad wavelengths (400 ​nm–700 ​nm) and limits light penetration into tissues.^59^ Competitive absorbance of melanin at certain wavelengths can reduce the efficiency of photosensitization by some types of photosensitizer, such as hypericin.^62^ Melanin is an effective quencher of singlet oxygen, which can protect melanocytes or melanoma cells from reactive oxygen species.^63^^,^^64^ As mentioned above, ROS is the main product of photodynamic therapy and melanin can reduce the efficiency of photosensitization as ROS scavenger. Depletion of melanin in vitro increased the peroxidation of membrane phospholipids and then enhanced the cytotoxicity of hypericin photodynamic therapy.^62^ Interestingly, melanosomes could be a target for photodynamic therapy as they are lysosome derived.^65^

In the clinic, verteporfin has been used to treat small choroidal melanoma. Pigmented tumors showed less tumor regression compared to the unpigmented tumors after photodynamic therapy with verteporfin: in one study, 53 out of 83 pigmented tumors showed complete or partial regression, whereas all 69 nonpigmented tumors showed complete regression.^24^

#### 4.1.3 Pigmentation and photodynamic therapy in cancer therapy, specific ocular tumors

We tested the influence of pigmentation on the treatment with Bel-sar in vitro and in vivo using cell lines derived from B16F10: we studied the normal pigmented cell line as well as a B16F10 tyrosinase knock-out cell line.^66^ Knockout of the tyrosinase gene led to loss of pigmentation and immature melanosomes (stage I and II). We noticed that pigmentation did not affect in vitro binding or accumulation of Bel-sar. There may be two reasons. First, the pigmentation level or stage III or IV melanosomes may do not induce the binding and uptake of the drug. Second, the targeting ability and high active molecule load of Bel-sar contributed to accumulation of PS at the tumor. Bel-sar treatment induced near complete cell death in the pigmented as well the non-pigmented cell lines in vitro. In a murine in vivo model, Bel-sar treatment similarly inhibited tumor growth in the pigmented and non-pigmented model. Bel-sar overcame the resistance mechanism of PDT in melanoma. It may due to the unique characteristics of Bel-sar itself, as described above. However, partial destruction of the tumor and tumor regrowth are still the greatest challenge in photodynamic therapy. Multiple treatments or the combination with other therapies, such as immunotherapy, may be necessary to induce complete tumor ablation in further pre-clinical or clinical trials.

#### 4.1.4 Conjunctival melanoma

Conjunctival melanoma (CJM) is a rare and malignant ocular tumor. After the first line treatment by excision combined with cryotherapy to the margins, 50% of patients may develop a local recurrence and 26% of patients develop metastases.^6^ We investigated the immunostimulatory ability of Bel-sar in conjunctival melanoma cells. When looking at the intracellular uptake of Bel-sar in CJM cells, we saw that after binding to the membrane, the VLPs are engulfed into the intracellular space and are mainly located in the lysosome, Mitochondria and Golgi.^67^ The three different CJM cell lines were all effectively killed after treatment with light-activated Bel-sar in vitro, indicating that Bel-sar treatment is a promising approach for this rare malignancy.

## 5 Biodistribution of bel-sar and its off-target effects

The use of VLPs for drug delivery has three key advantages: it limits off-target effects and may give rise to higher local concentrations.^33^ Most VLPs are not tumor tissue specific and drug accumulation is due to enhanced permeability: neovascularization allows preferential extravasation of particles of 40–120 ​nm to passively accumulate within a tumor, an observation referred to as the enhanced permeability and retention effect.^70^^,^^71^ Bel-sar preferentially leaks into the neo vasculature, and due to its tumor targeting capability, accumulates and specifically binds to HSPGs enriched on tumor cells, with a peak signal around 12 ​h after intravenous administration and a low signal in other organs 96 ​h after injection.^26^^,^^28^^,^^72^ In an intraocular orthotopic tumor model, an intravenous injection of Bel-sar and light treatment induced tumor necrosis with minimal damage to optical structures.^27^ It indicated that Bel-sar was mainly distributed to the eye tumor instead of other tissues of the eye. In another pre-clinical study using an orthotopic tumor model, a suprachoroidal injection induced higher Bel-sar accumulation compared with an intravitreal injection.^26^ In ongoing Phase II and III clinical trials, suprachoroidal administration of Bel-sar is being used to increase its distribution in the intraocular tumor and reduce any off-target effects. However, Bel-sar can also bind to other cells, such as epithelial cells or endothelial cells, and immune cells. Kines et al. showed that VLPs may bind to damaged epithelial cells.^25^ We observed that in a culture system, Bel-sar displayed a preference for cancer cells over dendritic cells.^72^ In Kines's studies, VLPs bound and were taken up by antigen-presenting cells of the host's immune system.^73^ In human peripheral blood leukocytes, the VLPs preferentially bind to neutrophils, monocytes, macrophages, dendritic cells and B cells, but not to T or NK cells.^74^ We know that UM contain populations of suppressive immune cells, such as tumor-associated macrophages and myeloid-derived suppressor cells, primarily serving to shield the tumor from host immune responses.^75^ Bel-sar can bind and may preferentially kill these phagocytic cells within the tumor microenvironment, giving further credence to its ability to generate a potent immunogenic tumor microenvironment. Based on this evidence, we can conclude that Bel-sar is selectively distributed to tumor tissue rather than normal tissues, and thereby mainly targets tumor cells. In addition, Bel-sar may be able to target immune cells which may contribute to the generation of a potent pro-immunogenic tumor microenvironment.

## 6 Anti-tumor efficiency: cytotoxicity, DAMPs and immunogenic cell death, immune responses initiated by bel-sar treatment

### 6.1 Intracellular and subcellular location of bel-sar, cell death

Photodynamic therapy is a clinically-approved cancer therapy, based on a photochemical reaction between a light-activatable molecule or photosensitizer, light and molecular oxygen. When these three otherwise harmless components are presented together, reactive oxygen species (ROS) are formed. Singlet oxygen is highly reactive, with a lifetime in the order of 40 ns and a maximum action radius of about 20 ​nm.^76^ This short action radius together with localized photosensitizer activation (only affecting illuminated target tissues) means that the localization of the photosensitizer influences the site of action of photodynamic therapy at the subcellular level.

We evaluated the cellular location of bel-sar in vitro at different time points. First, we observed that Bel-sar is located in the membrane and subsequently moves intracellularly. Similar to the molecular mechanism of HPV infection in keratinocytes,^77^ the VLPs first appear in the endosome or lysosome (4 ​h incubation, conjunctival melanoma cell line CRMM2). After 24 ​h of incubation, the VLPs are present in the Golgi and in mitochondria. It is known that the reaction of singlet oxygen with membrane lipids will result in lipid peroxidation and can lead to disruption of cellular membranes, resulting in the release of intracellular molecules.^78^ Targeting the lysosome, Golgi and mitochondria may help enhance tumor immunogenicity by inducing pyroptosis and preventing autophagy.^79^, ^80^, ^81^ This needs further investigation.

Traditionally, photodynamic therapy mainly induced three types of cell death: apoptosis, necrosis and autophagy.^46^ In the current study, light-activated Bel-sar induced apoptosis and necrosis in ocular melanoma cells. Photosensitizers that localize to mitochondria are most likely to induce apoptosis,^82^ while when the photosensitizer is located in the plasma membrane, necrosis is induced. Disruption of the plasma membrane leads to leakage of intracellular material to the extracellular matrix which can cause inflammation.^83^ The subcellular location of a photosensitizer has been associated with the type of cell death and the release of damage-associated molecular patterns (DAMPs).^46^

### 6.2 DAMPs and immunogenic cell death

**Fig. 2:**
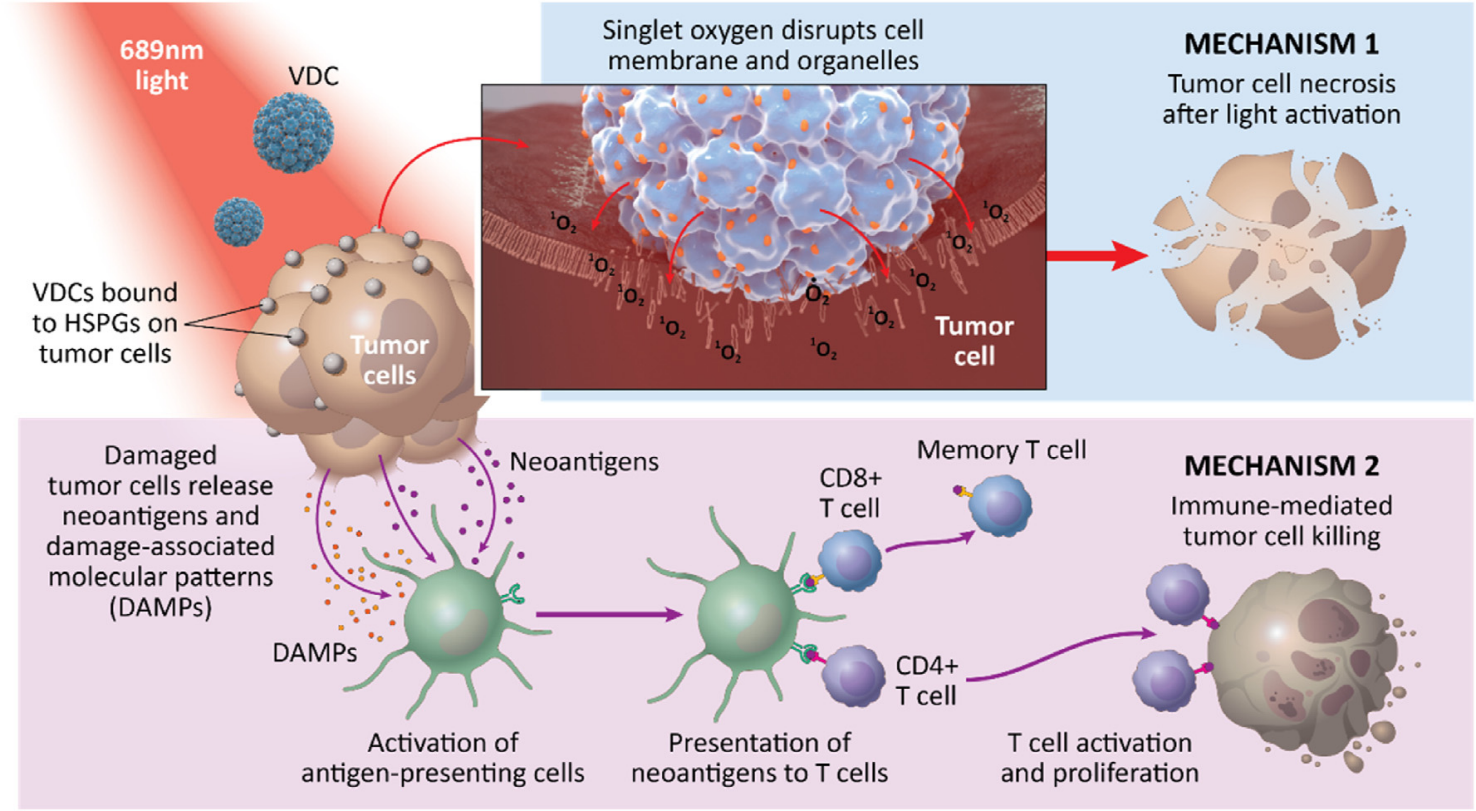
*The antitumor mechanism of light-activated Bel-sar in cancer therapy.* VDCs specifically bind to the HSPGs on the tumor membrane and then co-locate in the membrane and organelles. In the presence oxygen, laser activates the photosensitizer, initiating a photochemical reaction. This results in the generation of reactive oxygen species (ROS) within tumor cells. ROS can kill tumor cells directly, and then lead to exposure and release of damage-associated molecular patterns (DAMPs). These DAMPs and neoantigens stimulate or activate antigen presenting cells), which then present antigenic peptides to effector T cells, resulting in an adaptive immune response directed against tumor cells.

As mentioned above, photodynamic therapy does not only lead to direct cell death, but in addition, can initiate immunogenic cell death, a mode of cell death that is often accompanied by the exposure or release of DAMPs. DAMPs induced by photodynamic therapy include calreticulin (CRT), high mobility group box (HMGB1), and heat shock proteins (HSPs). HSP70 and HSP90 have been shown to induce maturation of antigen-presenting cells) and are able to initiate an immune response (Fig. 2).^19^^,^^20^^,^^84^ Light activation of Bel-sar induced immunogenic cell death in uveal melanoma cell lines, characterized by enhanced membrane exposure of CRT and HSP90. The effect was higher in BAP1-positive UM cell lines, with a lower IC_50_ and higher CRT exposure (Table 1). Pigmentation levels did not affect the exposure of DAMPs as similar levels were observed when pigmented and unpigmented B16F10 ​cells were treated with light-activation of Bel-sar. Bel-sar-treated tumor cells enhanced phagocytosis by antigen-presenting cells and stimulated the maturation of dendritic cells, increasing the expression of CD86 and MHC-II (Table 2). Mature dendritic cells subsequently migrated to tumor-draining lymph nodes and presented antigens to cytotoxic T cells, initiating a systemic immune response. These data indicate that the cell death triggered by PDT is immunogenic and PDT could be used as an adjuvant to initiate a strong local acute inflammation and a systemic immune response.^21^^,^^22^^,^^66^

Similarly, Bel-sar treatment was effective against three CJM cell lines in vitro and enhanced both the membrane exposure of DAMPs as well as the engulfment by macrophages (Table 1). One can imagine that Bel-sar treatment could serve as a local immune adjuvant to reduce CJM recurrences.

### 6.3 Local and systemic immune response

Immunogenic cell death induced by PDT could trigger a series of immunological events at different time points, such as a local inflammatory response at the treated site, which can develop into systemic antitumor immunity, providing long-term tumor control. Especially an immunologically “cold tumor’’ such as a uveal melanoma harbors an immunosuppressed microenvironment. The low mutational burden of UM and the immune privilege of the eye may make the intraocular tumor quite resistant to immune therapy.^15^^,^^85^ The onset of induced inflammation is marked by dramatic changes in the tumor vasculature that becomes permeable to blood proteins and pro-adhesive for inflammatory cells.^86^ Together with DAMPs, these lead to rapid infiltration of neutrophils, mast cells and monocytes/macrophages into the tumor site to remove cell debris.^21^^,^^87^ Such infiltrating immune cells could overcome the local immune suppression.

Macrophages constitute an important type of infiltrating immune cell in malignancies and are considered to be highly plastic cells, which can be activated in response to different signals.^88^ In UMs, high numbers of mainly M2 macrophages accompany heavy pigmentation and are associated with the development of metastases.^89^, ^90^, ^91^ Macrophages have various functions: M1-type macrophages are able to destroy the tumor structure by phagocytosis, eliciting vascular damage and tumor necrosis, whereas M2-type macrophages have the potential to enhance angiogenesis and induce immune suppression.^92^ Photodynamic therapy may lead to polarization of M2 macrophages into M1 macrophages, which could overcome a local immunosuppressive environment. Kines et al. observed that Bel-sar preferably binds to tumor-associated macrophages than to other immune cells.^73^ It is known from other studies, that Bel-sar bound to M2 macrophages rather than M1 macrophages and killed the M2 macrophages.^73^^,^^93^ When we treated pigmented and non-pigmented B16F10 subcutaneous tumors in mice with Bel-sar and light, it triggered infiltration with especially M1 macrophages, while reducing the number of M2 macrophages in both a pigmented and a non-pigmented tumor model.^66^ We furthermore saw that Bel-sar treatment induced the maturation of DCs. We also saw an induction of IFN-gamma positive CD8^+^ T cells in the tumor as well as in the draining lymph nodes, but this occurred only in the pigmented tumor model.

The biggest challenge of PDT is a partial, incomplete tumor ablation that may result in tumor cell survival after treatment, followed by rapid regrowth and tumor progression. Tumor killing may be improved by combining PDT with immunotherapy, such as immune checkpoint inhibitors, to enhance the systemic immune response against the tumor. Several studies have combined PDT with ICI antibodies to enhance the antitumor efficacy of PDT, which is mainly CD8+T cell dependent.^94^^,^^95^ When immunogenic cell death triggered by PDT is synergized with the induction of tumor-specific T cells through the use of immune checkpoint inhibitors, this combination may be able to function as an in situ vaccination strategy in cancer treatment. We compared the efficacy of combining Bel-sar with several different immune checkpoint inhibitor antibodies to identify optimal treatment regimens in a mouse tumor model (MC38, subcutaneous).^72^ The addition of immune checkpoint inhibitors (directed against CTLA-4, PD-L1 and LAG-3) enhanced the efficiency of Bel-sar against established tumors in mice, resulting in complete responses in all treated animals bearing a single tumor. Even more interesting, the combination of Bel-sar plus anti-PD-L1/anti-LAG-3 antibody treatment led to an optimal combination in an abscopal model, including complete responses of distant, non-illuminated tumors. The distant tumor ablation indicated that a strong systemic immune response was established. These results could be explained by prior observations that anti-PD-L1 treatment induces the expansion of tumor infiltrating CD4^+^ and CD8^+^ T cell subsets that co-express activating (ICOS) and inhibitory (LAG3, PD-1) molecules.^96^

## 7 Conclusions

A new drug, Bel-sar, made up of Virus-like particles conjugated with the photosensitizer phthalocyanine, is being used to treat small choroidal melanoma in order to induce in situ tumor ablation and preserve vision. VLPs derived from HPV-16 harbor natural targeting abilities to many cancer types and are an effective vehicle to deliver a large number of photosensitizer molecules to the tumor. The photochemical reaction is initiated only when tumors are irradiated with the appropriate infrared wavelength. These characteristics minimize the off-target effects and enhance drug accumulation in tumors. In experimental models, Bel-sar was shown to be an effective treatment to induce immunogenic cell death regardless of genetic heterogeneity and pigmentation level. Suprachoroidal injection of VDCs administered in multiple treatments is currently in clinical trials for the induction of complete tumor ablation. In our murine model, immune checkpoint inhibition combined with light-activated VDCs induced complete responses in local as well as distant tumors. Patients with not only ocular but also other malignancies could benefit of Immuno-PDT combination strategies, which will be the subject of future clinical studies.

## Study approval

Not Applicable.

## Author contributions

SM: conceptualization, design, writing original draft and revision; ​R.V.H.i.‘t.V: Writing – original draft preparation; E.D.L.P, F.A.O and M.J.J: conceptualization, supervision, manuscript review, and editing. ​All authors reviewed the results and approved the final version of the manuscript.

## Funding

This work was supported by a grant from Health Holland grant to M.J.J and Aura Biosciences (No. LSHM19061).

## Declaration of competing interest

The authors declare that they have no known competing financial interest or personal relationships that could have appeared to influence the work reported in this paper.

## Abbreviations

BAP1: BRCA1-associated protein-1
Bel-sar: belzupacap sarotalocan
CRT: calreticulin
CJM: conjunctival melanoma
CPMTV: cowpea mosaic virus
DAMPs: damage-associated molecular patterns
HMGB1: high mobility group box
HPV: human papillomavirus
HSP: heat shock proteins
HSPGs: heparin sulfate proteoglycan
ICIs: immune checkpoint inhibitors
NPs: nanoparticles
ROS: reactive oxygen species
UM: uveal melanoma
VDC: virus-like drug conjugate
VLPs: virus-like particles
